# Uniaxial tensile material properties of adult Chinese dura mater: investigating the influence of age, sex, and anatomical site

**DOI:** 10.3389/fbioe.2025.1550228

**Published:** 2025-07-29

**Authors:** Jinming Wang, Changsheng Cai, Lei Wan, Ao Liu, Kaifei Deng, Ying Fan, Jianhua Zhang, Jiang Huang, Changwu Wan, Donghua Zou, Zhengdong Li

**Affiliations:** ^1^ Department of Forensic Medicine, School of Basic Medical Sciences, Fudan University, Shanghai, China; ^2^ Shanghai Key Laboratory of Forensic Medicine, Shanghai Forensic Service Platform, Key Laboratory of Forensic Science, Ministry of Justice, Academy of Forensic Science, Guiyang, China; ^3^ School of Forensic Medicine, Guizhou Medical University, Guiyang, China; ^4^ Department of Radiology, Shanghai Public Health Clinical Center, Fudan University, Shanghai, China; ^5^ School of Electrical and Mechanical Engineering, Hunan University of Science and Technology, Xiangtan, China

**Keywords:** dura mater, biomechanics, material properties, craniocerebral injury, forensics

## Abstract

**Objective:**

This study investigated the biomechanical properties of the dura mater from 29 Chinese adult donors (20 -86 years), focused on the influence of age, anatomical region, sex and loading direction, to establish Chinese population - specific material parameters for cranial finite element (FE) models and enhance forensic traumatic brain injury analysis.

**Methods:**

In this study, a total of 275 dural specimens were prepared and categorized into young adult (20-44 years), middle aged (45-64 years), and elderly (≥65 years) cohorts. Samples were excised from frontal, temporal, parietal and occipital regions and tested uniaxially in sagittal and coronal directions, with strain measured via digital image correlation (DIC) techniques. True stress-strain curves were fitted to the Raghavan model to determine elastic fiber modulus (*E_E_*), collagen fiber modulus (*E_C_*), failure stress (*σ_Tf_*), and failure strain (*ε_Tf_*); ultimate tensile force (MaxForce) was also recorded. Histological analysis assessed age-related microstructural changes.

**Results:**

indicated significant age-related degradation: *E_C_*, *σ_Tf_*, *ε_Tf_*; and MaxForce significantly decreased with age (median *E_C_* declined from 28.0 MPa in young adults to 15.3 MPa in the elderly, P < 0.05; median *ε_Tf_* from 0.215 to 0.156, P < 0.05), while *E_E_* showed no significant age correlation (P = 0.10). Significant regional variance were observed, with the parietal region exhibiting higher *E_E_* (P = 0.01) and *σ_Tf_* (P = 0.03) compared to the occipital region; *ε_Tf_* showed no significant regional differences (P = 0.12). Dura mater demonstrated clear anisotropy: sagittal loading yielded significantly higher median *E_C_* (27.0 MPa vs. 18.1 MPa coronal, P = 0.003), *σ_Tf_* (4.30 MPa vs. 3.18 MPa coronal, P = 0.020), and MaxForce (12.9 N vs. 10.3 N coronal, P = 0.014). No statistically significant sex-based differences were found for any parameter (P > 0.05). Histology confirmed progressive age-related collagen disorganization and elastic fiber degradation. In conclusion, Chinese adult dura mater exhibits significant age-dependent decline in mechanical integrity, clear anisotropy favoring the sagittal direction, and notable regional heterogeneity, but no significant sex-based differences.

**Conclusion:**

These findings provide crucial, population-specific data for improving the biofidelity of FE head models and forensic injury analysis.

## 1 Introduction

Understanding the injury mechanism underlying craniocerebral trauma remains a critical challenge in forensic practice, necessitating urgent advancements in analytical approaches. Traditional forensic methods for identifying craniocerebral trauma mechanisms have predominantly relied on injury pattern analysis and subjective expert assessment, which often results in largely qualitative determinations that may not adequately capture the quantitative relationships between injurious loading conditions and the resultant pathological outcomes. This inherent limitation presents a significant obstacle to achieving precise and objective forensic injury identifications (Jia et al., 2020; Xu et al., 2023).

With advances in computational science and digital simulation technology, finite element modeling (FEM) of the craniocerebral system has emerged as a powerful and increasingly predominant research approach in forensic traumatology. FEM allows for the reconstruction of traumatic brain injury (TBI) processes and detailed investigation of associated injury mechanisms. These computational models enable the detailed analysis of brain tissue stress-strain responses under diverse trauma scenarios, thereby providing crucial insights into the biomechanical factors contributing to craniocerebral injury causation (Sha et al., 2010).

Despite their widespread adoption and utility, current finite element head models possess notable limitations that can affect their predictive accuracy. Key limitations include the insufficient characterization of region-specific material properties for cranial tissues, particularly the dura mater, and often oversimplified representations of regional brain tissue structural complexities. Such deficiencies directly impact biofidelity of these models, particularly concerning the accuracy of material parameter assignments and the definition of region-specific injury thresholds. Research has consistently demonstrated that the dura mater not only significantly influences overall skull biomechanical responses but also plays an essential protection role during dynamic loading events, such as by mitigating brain compression (Chen et al., 2016; Kizmazoglu et al., 2019; Ondruschka et al., 2019). Therefore, developing more anatomically faithful and materially accurate representations of dura mater within craniocerebral finite element models is crucial for advancing our understanding of TBI mechanisms (Miller et al., 2016).

Uniaxial tensile testing remains the primary method for characterizing the macroscopic material properties of the dura mater, valued for its simplicity and established reliability in testing soft biological tissues. Numerous recent studies have employed this approach to extensively investigate human dura mater properties (Table 1). For instance, Li et al. (2020) reported an overall tensile strength of 9.79 ± 3.39 MPa for frontal dura mater failure stress in Chinese populations aged 20 years, a value that differs from some data reported for other populations. Comparatively, studies by Zwirner et al. (2019) reported that the tensile strength of human temporal dura mater is 7 ± 4 MPa and Zwirner et al. (2019) also reported a value of 7.01 ± 0.77 MPa for dura mater, respectively, leading further support to the possibility of population-specific biomechanical variations. However, significant variability in reported data exists across the literature. This is often attributed to a lack of standardization in experimental protocols, including differences in specimen procurement, preparation (e.g., dimensions), testing methodologies, and data analysis, all of which can contribute inconsistent outcomes and impede the establishment of unified reference standards.

**TABLE 1:** Summary of the test results of the published uniaxial tensile tests of dura mater.

Author	Specimen	Morphology	Dimension	Device	Direction	Sample size	Failure stress (MPa)	Failure strain
Patin et al. (1993)	Dura mater	—	10 mm × 15 mm	InstroModel 1000, Canto, MA	T, L	7	—	—
Kizmazoglu et al. (2019)	Dura mater	Dogbone-shaped	60 mm	AG-IS 5 kN, Shimadzu Corporation, Kyoto, Japan	—	10	7.01 ± 0.77	—
Zwirner et al. (2019)	Dura mater	Dogbone-shaped	20 mm	LTM10, ZwickRoell; CCD camera, ZwickRoell, Ulm, Germany	—	73	7 ± 4	11% ± 3%
Li et al. (2020)	Dura mater (Frontal)	Square shape	40 mm × 10 mm	KDII 0.2	—	284	10.39 ± 2.94	—
Pearcy et al. (2022)	Dura mater (Frontal)	Dogbone-shaped	20 mm × 10 mm	Allround Table Top Z020 with an Xforce P load cell of 2.5 kN with test-Control II measurement electronics, Zwick Roell, Ulm, Germany	T, L	244	11.1	—
Cavelier et al. (2022a)	Dura mater	Square shape	36 × 5 mm	5543, Instron, High Wycombe, UK	T, L	7	9.76	—
Niestrawska et al. (2023)	Dura mater	Dogbone-shaped	20 mm	Nominal force 500 N; (ZwickRoellAG, Ulm, Germany) Aramis image correlation system	T, L	5	4.49	1.10%
Zwirner et al. (2023)	Dura mater	Dogbone-shaped	20 mm × 5 mm	CCD camera, ZwickRoell, Ulm, Germany; LTM10, ZwickRoell	T, L	30	5.16	—

Note: T is Transversal, corresponds to the coronal direction; L is Longitudinal, corresponds to the sagittal direction.

The biomechanical properties of the dura mater are also known to exhibit regional heterogeneity. Niestrawska et al. (2023) recently provided compelling evidence linking location-dependent variations in collagen fiber orientation and dispersion directly to differences in local mechanical anisotropy. Their morpho-mechanical mapping demonstrated that main fiber angles and tissue stiffness varied significantly across different cranial locations. Early work by Patin et al. (1993) observed directional differences in dura mater properties, noting higher stiffness and strength along the sagittal suture compared to the coronal suture. Conversely, other studies, such as De Kegel et al. (2018), have reported no statistically significant regional variations in failure stress, highlighting the ongoing debate and the need for further comprehensive investigations. Pearcy et al. (2022) introduced another important consideration, revealing that the vascular presence and orientation within the dura mater significantly influence mechanical properties, with transverse vessel-containing dura exhibiting reduced tensile strength (11.1 MPa) compared to longitudinal vessel specimens (15.0 MPa). While such findings related to vessel orientation suggest a form of structural directionality, the overall mechanical anisotropy of the dura mater is primarily governed by its collagen fiber architecture. Histological evidence often indicates complex, non-orthogonal fiber arrangements, resulting in relatively modest directional effects on the tissue’s mechanical response.

Furthermore, many existing studies have focused on characterizing the mechanical properties from only a single anatomical region or a limited regions, thus restricting comprehensive direct comparisons of regional heterogeneity. Moreover, a significant scarcity of comprehensive data persists specifically for the biomechanical properties of dura mater from the Chinese population, which constrains the development of population-specific craniocerebral finite element models and impedes advancements in forensic biomechanical analyses tailored for this demographic. Addressing this gap necessitates a systematic and comprehensive investigation of dura mater biomechanical properties across various influencing factors within the adult Chinese population.

The present study employs an Instron 8,874 (Illinois Tool Works Inc., United States) testing system equipped with digital image correlation (DIC) for precise strain measurements. We will comprehensively characterize key dura mater material parameters—including failure stress, failure strain and elastic modulus—from adult Chinese subjects. These parameters will be investigated across different age groups, anatomical regions, testing directions and sexes. This investigation aims to generate an essential dataset for material properties specific to the adult Chinese population and improve material parameter assignments in Chinese-specific craniocerebral FEM, ultimately enhancing the accuracy of trauma simulations and advancing the capabilities of forensic biomechanical analysis (Barbeito-Andres et al., 2020; Alshareef et al., 2025).

## 2 Materials and methods

### 2.1 Sample collection

This study was performed in accordance with the Declaration of Helsinki and received ethical approval from the Ethics Committee of the Academy of Forensic Science, Ministry of Justice, China (Approval No. 2022-7). The approved protocols encompassed both sample collection from deceased donors and the procedures for obtaining informed consent from next-of-kin for the use of samples in research. The study utilized fresh dura mater samples from 29 donors obtained from forensic autopsy cases at the Academy of Forensic Science. Specimens were coded as C1 to C29 for anonymization and age was recorded for each donor. Sample selection adhered to the following strict inclusion criteria (Samsell et al., 2019; Li et al., 2023): (Jia et al., 2020) samples extracted from cadaver donors that were either maintained at ambient temperature for a maximum of 24 h or preserved under freezing conditions at −20°C for no more than 7 days; (Xu et al., 2023); cases involving open craniocerebral trauma were excluded; and (Sha et al., 2010) specimens exhibiting any structural damage, necrotic changes, morphological abnormalities, or pathological alterations identified during autopsy were omitted. The study population encompassed an age range of 20–86 years. All samples were collected and processed as rapidly as possible within these stipulated postmortem timeframes and conditions to minimize potential degradation.

### 2.2 Specimen classification and preparation

**FIGURE 1:**
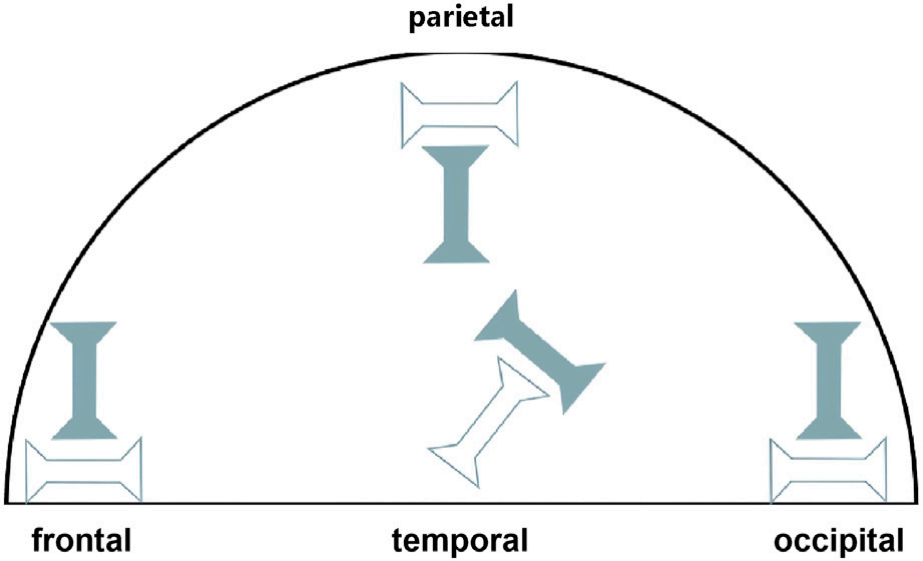
Schematic diagram of dural sampling locations from the frontal, temporal, parietal and occipital regions. Hollow symbols indicate samples excised along the sagittal direction, and solid symbols indicate samples excised along the coronal direction.

The collected dura mater specimens were stratified into three age cohorts: young adult (20–44 years; n = 10), middle-aged (45–64 years; n = 10) and elderly (≥65 years; n = 9), with further subdivision by donor sex within each cohort. These age groupings were revised from our initial approach to create cohorts with more comparable age spans, thereby addressing concerns about group interval uniformity, while maintaining balanced sample sizes for statistical comparisons. These classifications are also broadly consistent with life stage categorizations used in medical research (Khera et al., 2017; Choi et al., 2023). The overall anatomical orientation of each dural sheet was first noted. Test specimens were then prepared along two predefined testing directions: parallel to the sagittal plane (sagittal direction) and parallel to the coronal plane (coronal direction). Using a customized 6 mm-wide die, dogbone-shaped test specimens (Khera et al., 2017; Choi et al., 2023) were excised from four distinct anatomical regions: frontal, temporal, parietal and occipital (see Figure 1 for sampling locations). These four regions were chosen as they represent the major expanses of the cranial dura overlying the principal brain lobes, are commonly investigated in dural biomechanics literature (Cavdar et al., 2022; Zwirner et al., 2021), and are relevant to common TBI impact scenarios encountered in forensic investigations. A total of 275 specimens were prepared for tensile testing.

**FIGURE 2:**
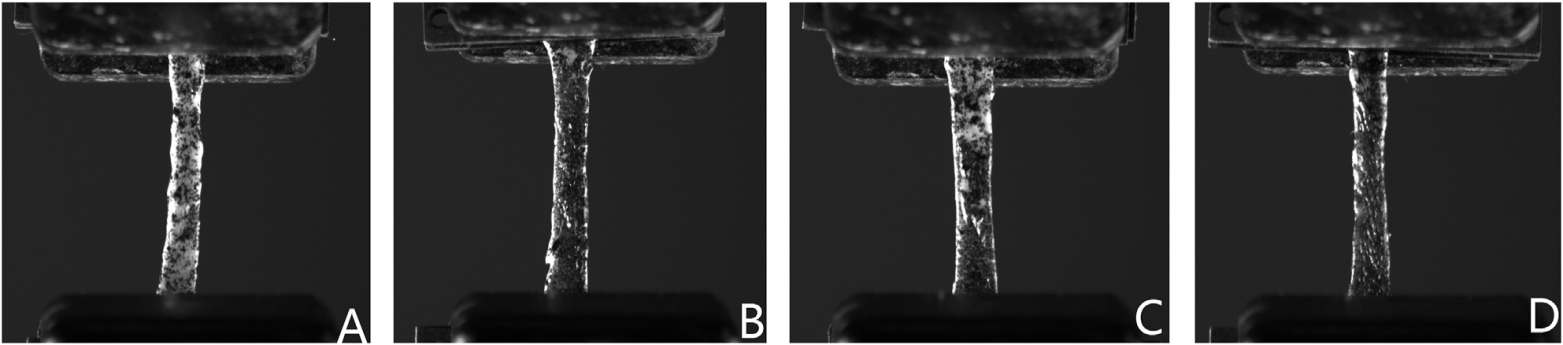
Representative DIC images of human dura mater specimens under uniaxial tension. Images show typical speckle patterns applied for strain tracking and illustrate specimen deformation, including some necking phenomenon observed prior to failure, from **(A)** frontal, **(B)** temporal, **(C)** parietal, and **(D)** occipital regions.

The test area of the specimen was photographed with a customized profile measurement system (Li et al., 2023) and the specimen thickness was measured at three points along the gauge length (both ends and the center) with VisionMaster3 software (Hikrobot Co., Ltd., China; resolution 0.011 mm). All specimen dimensional measurements (thickness) were performed by a single trained researcher following a standardized protocol to ensure consistency. The arithmetic mean of these measurements was recorded as the specimen’s thickness (Supplementary Table 1). For non-contact strain measurement using DIC, a fine mist of black matte paint was sprayed on the gauge area of each specimen using an adjustable spray gun (0.3–0.6 MPa), which aimed to create a random, high-contrast speckle pattern with speckles of a visually optimal size for subsequent DIC tracking. To enhance gripping and prevent slippage, a thin layer of cyanoacrylate adhesive was applied to the gripping areas at the extremities of each specimen outside the gauge length, and allowed to cure before clamping. To maintain tissue hydration throughout the testing process, specimens were kept moist with physiological saline (0.9% NaCl solution), applied by spraying or dripping as needed. The axial elongation of the gauge section of the specimens during tensile testing was monitored in real time using a DIC system (resolution 5 M pixels), which provided precise measurements of gauge length change (*ΔL*) for calculating engineering strain for the subsequent analysis (Figure 2).

### 2.3 Mechanical testing and parameter assessments

To minimize operator-dependent variability, all uniaxial tensile tests and DIC procedures were performed by a single, experienced researcher. Uniaxial tensile tests were conducted at ambient temperature (approximately 25°C ± 4°C) using an Instron 8,874 universal testing system (Illinois Tool Works Inc., United States). Specimens were carefully mounted in the grips of the testing machine in a natural, untensioned state, ensuring alignment of the specimen’s longitudinal axis with the loading axis of the machine. The initial gauge length (*L*_*0*_), defined as the distance between the points tracked by the DIC system on the specimen surface within the gauge section, was recorded after a minimal pre-load (0.2 N) was applied to remove slack in the specimen. Prior to definitive testing, each specimen underwent five preconditioning cycles at 20% × *L*_*0*_/min with a 4% × *L*_*0*_ displacement amplitude to achieve a state of mechanical equilibrium and ensure repeatable mechanical responses (Pei et al., 2021).

Following preconditioning, definitive quasi-static uniaxial tensile testing was performed at a constant crosshead displacement rate of 5 mm/min until complete specimen failure. During testing, the system’s load cell and displacement transducer continuously recorded force and crosshead displacement data. From these, force-displacement relationships and ultimate tensile force (MaxForce) were directly obtained. Tensile strength was calculated post-test (see Section 2.4.1). The testing system records force-displacement relationships and ultimate load is derived. Concurrent strain measurements of gauge section elongation were obtained via the DIC system at a 1 Hz sampling frequency, enabling real-time data synchronization with the load cell. Strict adherence to a standardized protocol for specimen mounting, pre-load application, and DIC system calibration was maintained to enhance the consistency and reliability of mechanical measurements. DIC system calibration was performed before testing sessions using a 25 mm * 25 mm calibration plate with a precise 12 * 9 dot array (18 mm * 13.5 mm area, 0.01 mm pattern accuracy) to correct for lens distortions and determine image scaling factors.

### 2.4 Data analysis

#### 2.4.1 Stress-strain definitions and calculations

The analysis incorporated the initial dimensional characteristics (width and thickness) of each specimen. Dura mater was assumed to be incompressible during tensile deformation (Khera et al., 2017). This assumption of incompressibility enabled the calculation of true strain *ε*_*T*_ and true stress *σ*_*T*_) from engineering strain *ε*_*E*_ and engineering stress *σ*_*E*_.

The engineering strain (*ε*_*E*_) for the defined gauge section was defined as the change in gauge length (*ΔL = L-L*_*0*_) relative to the initial gauge length (*L*_*0*_), calculated by the following formula:

$$\varepsilon_{E}=\frac{L-L_{0}}{L_{0}},$$

Where *L*_*0*_ is the initial gauge length determined by the DIC system before tensile loading, and *L* is the current gauge length at a given point during deformation.

The engineering stress (*σ*_*E*_) represents the ratio of the applied tensile force (*F*) on the specimen relative to its initial cross-sectional area (*A*_*0*_), calculated using the following formula:

$$\sigma_{E}=\frac{F}{A_{0}},$$

*A*_*0*_ is the initial cross-sectional area, calculated from the mean specimen width (6 mm from the die) and the measured mean thickness (see Section 2.2).

True strain ( $\varepsilon_{T}$ ) takes into account the continuous change in length during the tensile of the samples and is calculated as:

$$\varepsilon_{T}=\ln\left(1+\varepsilon_{E}\right)$$

Assuming material incompressibility, true stress ( $\sigma_{T}$ ) was calculated from engineering stress using the following equation:

$$\sigma_{T}=\sigma_{E}\left(1+\varepsilon_{E}\right)$$

To characterize the non-linear stress-strain behavior of the dura mater, which reflects its composite nature, the experimental true stress-true strain curves for each specimen were fitted to the mathematical model proposed by Raghavan et al. (1996). This model describes the strain (*ε*) as a function of stress (*σ*) using the following relationship:

$$\varepsilon_{T}=\left(K+\frac{A}{B+\sigma_{T}}\right)\sigma_{T}$$

In this mathematical model, *K*, *A* and B are material constants determined by a least-squares fit of the experimental data up to the point of failure. These parameters have specific physical interpretations related to the tissue’s compliance at different stress levels (Raghavan et al., 1996). Parameter *K* represents the tissue’s compliance (inverse of stiffness) at very high stress levels ( $\sigma_{T}\;\to \infty$ ), primarily attributed to the response of fully engaged collagen fibers. The sum ( K+A/B ) represents the tissue’s compliance at very low stress levels (as $\sigma_{T}\;\to \;0$ ), predominantly governed by the behavior of elastic fibers. For this sum to represent initial compliance and be greater than *K* (final compliance), *A* and *B* typically share the same sign (usually positive). The parameter A (units of stress) contributes to defining the initial compliance and the shape of the transition region. The parameter *B* (units of stress) is a measure of the stress level at which the tissue transitions from its initial high compliance (elastin-dominated) state to a lower compliance (collagen-dominated) state. From these model parameters, two effective moduli can be derived to represent the stiffness of the dura mater at low and high strain regions, corresponding conceptually to the contributions of elastin and collagen, respectively.

The elastic fiber-associated modulus (*E*_*E*_), representing the stiffness in the initial, lower-stress, elastin-dominated response region, is calculated as:

$$E_{E}=\frac{\sigma }{\varepsilon }=\frac{1}{K+A/B}$$

The collagen-associated modulus (*E*_*C*_), representing the stiffness in the high-stress, collagen-dominated response region, is calculated as:

$$E_{C}=\frac{A}{K\left(A+KB\right)}$$

These derived moduli *E*_*E*_ and *E*_*C*_, along with the model parameters *K*, *A*, and *B*, provide a comprehensive characterization of the dura mater’s non-linear tensile behavior.

#### 2.4.2 Data processing and statistical analysis

All statistical analyses were performed using open source Jamovi (Version 2.3.12.0, The Jamovi Project, Sydney, Australia; jamovi.org). For each specimen, true stress-true strain curves were generated as described in Section 2.4.1. The true stress at failure (failure stress, *σ*_*Tf*_) and true strain at failure (failure strain, *ε*_*Tf*_) were determined from these curves, corresponding to the point of specimen fracture.

The experimental true stress-true strain data for each specimen (up to failure) were fitted to the Raghavan model (Raghavan et al., 1996) using a non-linear least-squares regression algorithm (Levenberg-Marquardt Algorithm) to determine the optimal parameters *K*, *A* and *B*. This non-linear regression was performed using a custom script developed in Python (version 3.10.11) utilizing the SciPy library’s curve_fit function. These fitted parameters were subsequently used to calculate elastic fiber modulus (*E*_*E*_) and collagen fiber modulus (*E*_*C*_) for each specimen.

Due to the observed dispersion in some datasets, descriptive statistics for all determined mechanical parameters (*E*_*E*_, *E*_*C*_, *σ*_*Tf*_, *ε*_*Tf*_, *K*, *A*, *B*, and the directly measured ultimate tensile force (MaxForce)) within each experimental group (defined by age cohort, anatomical region, and testing direction) were expressed as median (25th percentile, 75th percentile). This approach, along with the selection of statistical tests robust to non-normal distributions and variance heterogeneity (as detailed below), was chosen to appropriately manage the inherent biological variability observed among donors and among multiple specimens derived from individual donors. While each specimen was treated as a statistical unit for regional and directional comparisons, the use of robust descriptive and inferential statistics helps mitigate the influence of high inter-donor and intra-donor sample variability.

Prior to inferential statistical testing, data distribution was assessed for normality (using the Shapiro-Wilk test). For comparisons between independent groups (age cohorts, anatomical regions, male vs. female groups, sagittal vs. coronal testing directions), the Kruskal–Wallis H test (a non-parametric alternative to ANOVA) was employed. This test was used for comparisons involving two or more groups; for two-group comparisons, it is equivalent to the Mann-Whitney U test. If overall significance was found for comparisons involving three or more groups, *post hoc* pairwise comparisons were conducted using Dunn’s test with Bonferroni correction for multiple comparisons. To further assess trends with age as a continuous variable, Spearman’s rank correlation coefficient (*ρ*) was calculated between donor age and the key mechanical parameters. A p-value (P < 0.05) was considered statistically significant for all analyses.

### 2.5 Histological analysis

For histological examination, representative specimens were randomly selected from the three age cohorts (young adult: C2; middle-aged: C12; elderly: C26). From each of these three selected dural sheets (C2, C12, C26), tissue segments measuring approximately 1 mm × 3 mm were excised from areas representative of the mechanically tested regions (frontal, temporal, parietal, and occipital). These were used primarily to qualitatively assess and illustrate characteristic age-related microstructural changes across the different age cohorts. Tissue segments were fixed in 10% neutral buffered formalin (NBF) for 24 h, followed by processing through a standard ethanol dehydration series, clearing with xylene, and embedding in paraffin wax. Subsequently, the paraffin-embedded tissue blocks were sectioned at 3 μm thickness. Sections were mounted on glass slides, deparaffinized using xylene, and rehydrated through a graded ethanol series to distilled water. Following deparaffinization and rehydration with water, sections were oxidized with potassium permanganate solution for 5 min, followed by a brief water wash. Subsequently, sections were bleached with oxalic acid solution for 2–3 min until colorless and then briefly washed in water again. After a quick rinse in 95% ethanol, sections were immersed in the elastin staining solution (Verhoeff’s elastin stain) and incubated in a 37°C water bath for 30–60 min. The staining intensity and differentiation process were microscopically controlled. Differentiation was performed rapidly with 95% ethanol, ensuring excess stain was removed and carefully monitored under the microscope to prevent over-differentiation. Sections were then washed in running tap water for 2–3 min.

Counterstaining was performed with Van Gieson solution for 2–3 min. After decanting the excess solution, sections were rapidly differentiated with 95% ethanol. This staining protocol distinctly visualizes elastic fibers (stained blue-black) and collagen fibers (stained red). Nuclei typically appeared grey to blue (Kiernan, 2015). Stained sections were then dehydrated through graded alcohols, cleared, and coverslipped with a permanent mounting medium. Microscopic analysis was performed using a light microscope (Leica DM3100, Leica Microsystems, Wetzlar, Germany) to qualitatively assess the morphology, organization, and relative distribution of elastic and collagen fibers within the dura mater from different regions and age groups.

## 3 Results

### 3.1 Directional and age-related stress-strain response of dura mater under uniaxial tension

**FIGURE 3:**
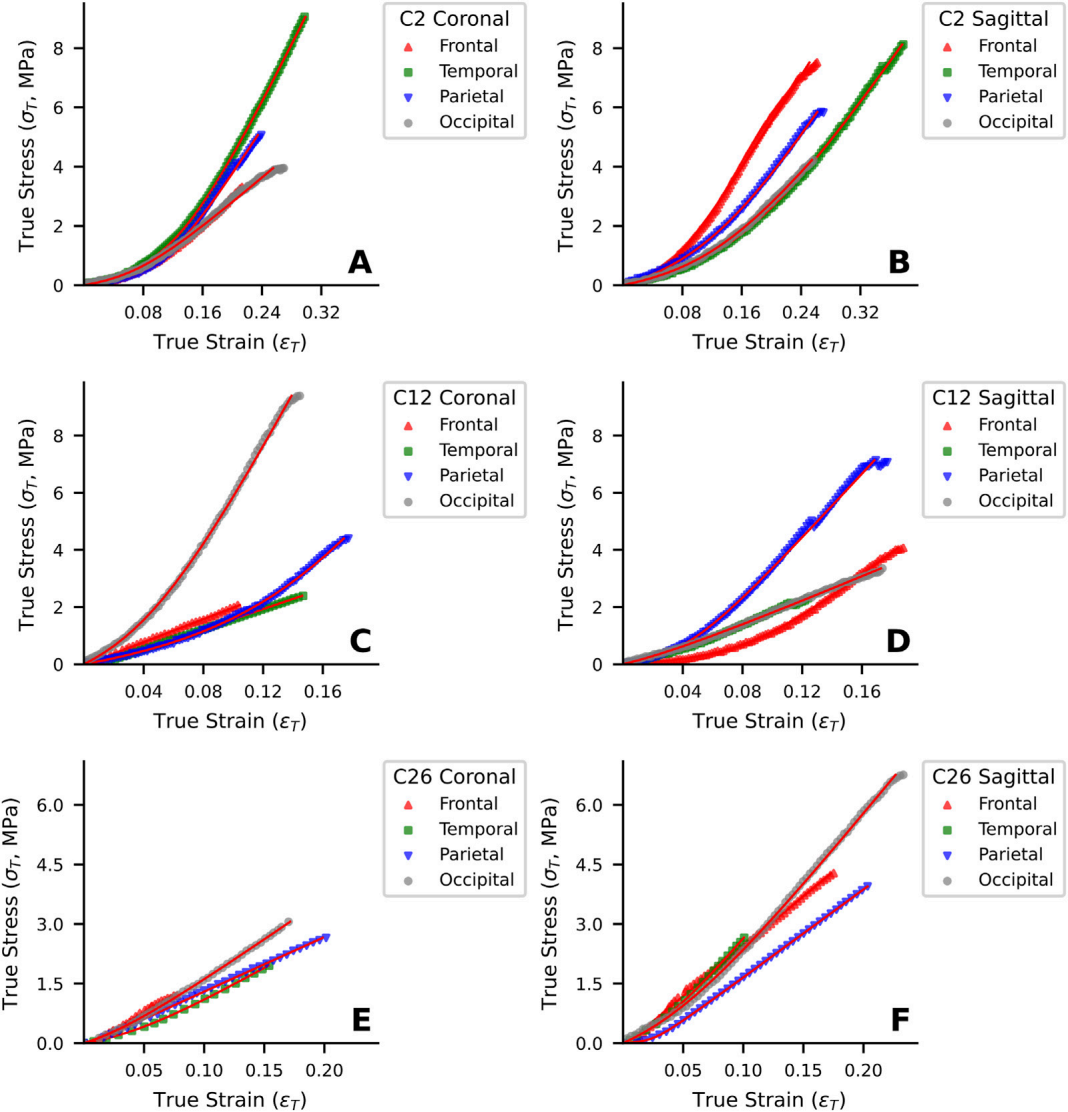
Representative uniaxial tensile stress-strain curves of human dura mater specimens from different age groups. **(A, B)** Specimen from a 25-year-old donor. **(C, D)** Specimen from a 48-year-old donor. **(E, F)** Specimen from a 76-year-old donor. Red lines represent the fitted curves.

The optimized testing protocol for human dura mater specimens yielded valid results for 232 out of 275 attempted tests (84.4%), with the 232 specimens failing in the middle portion of the gauge length. Specimens that failed at other locations were excluded from data analysis. The stress-strain behavior of the 232 specimens was characterized under uniaxial tensile loading in both coronal and sagittal directions across four anatomical regions (frontal, temporal, parietal and occipital) and stratified by age cohort. Representative stress-strain curves for specimens from different age groups (C2, C12 and C26) are presented in Figure 3. Visual inspection of the stress-strain curves (Figure 3) showed that specimens from all age groups typically exhibited a non-linear response with an initial region of notable stiffness, indicating resistance to deformation at low strains.

In the young adult cohort (20–44 years), the curves for coronal (Figure 3A) and sagittal (Figure 3B) loading directions appeared qualitatively similar, suggesting a tendency towards isotropic mechanism behavior in this group. In contrast, the stress-strain responses of middle-aged (45–64 years; Figures 3C, D) and elderly (≥65 years; Figures 3E, F) cohorts visually suggested more pronounced mechanical anisotropy. Specifically, for these older representative specimens, loading in the sagittal direction (Figures 3D, F) appeared to elicit higher stress responses for a given strain and higher failure stresses compared to the coronal direction (Figures 3C, E).

Young adult specimens (C2, Figures 3A, B) generally exhibited a distinct J-shaped curve, indicative of the organized engagement of fibrous components, though considerable regional and directional variations were apparent. In the middle-aged donor (C12, Figures 3C, D), the J-shaped response persisted, but with pronounced mechanical heterogeneity between regions and directions, suggesting early changes in tissue structural integrity. By contrast, elderly specimens (C26, Figures 3E, F) often showed a more linearized stress-strain response with a diminished “toe” region, particularly in the coronal direction, and typically reached lower failure stress and strain values. This altered mechanical signature in the elderly is consistent with the progressive microstructural degradation, such as loss of collagen organization and elastic fiber fragmentation, detailed in Section 3.6.

### 3.2 Parameter optimization and summary of material properties

The Raghavan material model, as defined in section 2.4.1, was fitted to each of the 232 experimental true stress-true strain curves. The parameter optimization process successfully converged for all specimens, yielding a unique set of fitting parameters (*K, A, B*) for each test. From these primary fitted parameters, the elastic fiber modulus (*E*_*E*_) and collagen fiber modulus (*E*_*C*_) were calculated respectively. These derived moduli, along with the failure stress (*σ*_*Tf*_), failure strain (*ε*_*Tf*_), and the directly measured ultimate tensile force (MaxForce), constitute the key mechanical properties evaluated in this study.

A summary of these material properties, stratified by age cohort, anatomical region, and loading direction, is presented in Table 2. For parameters not following a normal distribution (as determined by Shapiro-Wilk tests, P < 0.05), data are reported as median (25th percentile, 75th percentile).

**TABLE 2:** Summary of material properties stratified by age cohort, anatomical region, and loading direction.

Group	N	Region	Coronal direction					Sagittal direction				
			$E_{E}$ (MPa)	$E_{C}$ (MPa)	*σ*_*u*_ (MPa)	ε _*u*_	MaxForce (N)	$E_{E}$ (MPa)	$E_{C}$ (MPa)	*σ*_*u*_ (MPa)	ε _*u*_	MaxForce (N)
Young adult	10	F	6.33 (3.28, 8.42)	22.5 (9.61, 36.4)	3.39 (2.98, 6.27)	0.210 (0.159, 0.218)	13.6 (12.1, 17.6)	10.9 (5.81, 14.3)	41.5 (36.1, 48.7)	5.00 (3.80, 7.02)	0.186 (0.165, 0.247)	16.5 (13.8, 21.2)
		T	7.33 (5.02, 10.4)	18.8 (9.15, 35.7)	4.63 (3.23, 6.44)	0.250 (0.177, 0.285)	13.0 (8.48, 20.5)	9.60 (6.83, 11.8)	37.9 (33.0, 45.4)	5.91 (4.77, 6.99)	0.188 (0.160, 0.241)	19.2 (12.6, 20.4)
		P	5.06 (3.66, 10.6)	31.6 (20.6, 36.5)	5.71 (4.36, 6.86)	0.227 (0.190, 0.276)	16.6 (14.0, 19.2)	9.99 (7.71, 15.8)	34.9 (30.1, 44.4)	6.01 (4.68, 7.76)	0.215 (0.204, 0.225)	20.4 (15.8, 22.7)
		O	5.34 (4.36, 7.33)	16.9 (15.0, 21.3)	3.40 (2.72, 3.97)	0.233 (0.201, 0.284)	7.50 (6.85, 9.38)	3.54 (2.21, 5.18)	17.2 (11.8, 26.4)	3.85 (1.69, 5.91)	0.261 (0.225, 0.288)	8.45 (5.62, 15.2)
Middle-aged	10	F	5.97 (3.83, 17.1)	21.4 (12.4, 24.0)	3.41 (2.78, 4.16)	0.166 (0.118, 0.223)	10.5 (9.48, 13.6)	4.90 (3.22, 7.59)	21.4 (14.0, 31.5)	3.18 (2.20, 5.41)	0.182 (0.132, 0.209)	10.3 (7.35, 16.3)
		T	4.37 (2.34, 7.92)	16.0 (6.73, 36.3)	2.67 (1.88, 3.58)	0.159 (0.149, 0.182)	10.1 (6.03, 14.0)	7.35 (4.65, 10.2)	32.7 (15.5, 46.2)	3.75 (2.65, 5.79)	0.160 (0.124, 0.215)	10.9 (8.52, 15.6)
		P	10.2 (8.14, 16.8)	30.6 (28.7, 59.7)	4.79 (3.54, 6.59)	0.175 (0.127, 0.202)	15.9 (11.8, 17.2)	14.8 (9.47, 18.4)	28.3 (20.9, 44.0)	5.12 (4.31, 6.89)	0.173 (0.153, 0.190)	15.0 (12.2, 25.2)
		O	4.31 (3.28, 9.96)	23.0 (17.0, 38.6)	2.69 (2.19, 4.65)	0.157 (0.136, 0.218)	7.75 (6.25, 9.42)	5.43 (4.58, 11.0)	19.3 (10.7, 36.1)	2.99 (1.92, 5.75)	0.193 (0.152, 0.216)	8.90 (6.53, 12.4)
Elderly	9	F	0.833 (0.517, 5.90)	12.0 (8.66, 18.3)	1.84 (0.683, 2.43)	0.142 (0.077, 0.167)	6.80 (2.70, 7.50)	6.60 (1.28, 9.83)	24.8 (16.3, 34.4)	3.60 (1.45, 4.37)	0.132 (0.108, 0.175)	12.3 (5.10, 18.0)
		T	4.27 (2.07, 7.80)	9.59 (7.37, 15.2)	2.79 (1.95, 3.96)	0.226 (0.154, 0.260)	11.1 (7.60, 17.5)	13.0 (0.392, 23.1)	19.5 (11.0, 24.5)	3.57 (2.66, 4.84)	0.131 (0.108, 0.159)	11.0 (10.0, 26.9)
		P	7.79 (4.04, 12.5)	11.6 (10.5, 13.6)	2.17 (1.84, 2.65)	0.157 (0.131, 0.185)	7.50 (7.20, 11.1)	8.99 (0.288, 13.1)	33.1 (20.5, 53.4)	3.95 (3.52, 4.90)	0.148 (0.111, 0.187)	14.3 (9.80, 16.4)
		O	4.15 (3.08, 7.08)	12.5 (11.2, 15.0)	2.38 (1.07, 3.06)	0.170 (0.144, 0.213)	5.80 (4.90, 10.6)	4.51 (0.782, 9.89)	17.0 (8.36, 31.3)	3.56 (1.52, 5.36)	0.158 (0.124, 0.233)	11.7 (5.50, 12.9)

Note: Values are expressed as the Median (25th percentile – 75th percentile) (F, Frontal; T, Temporal; P, Parietal; O, Occipital).

### 3.3 Age-related variations in material properties

**FIGURE 4:**
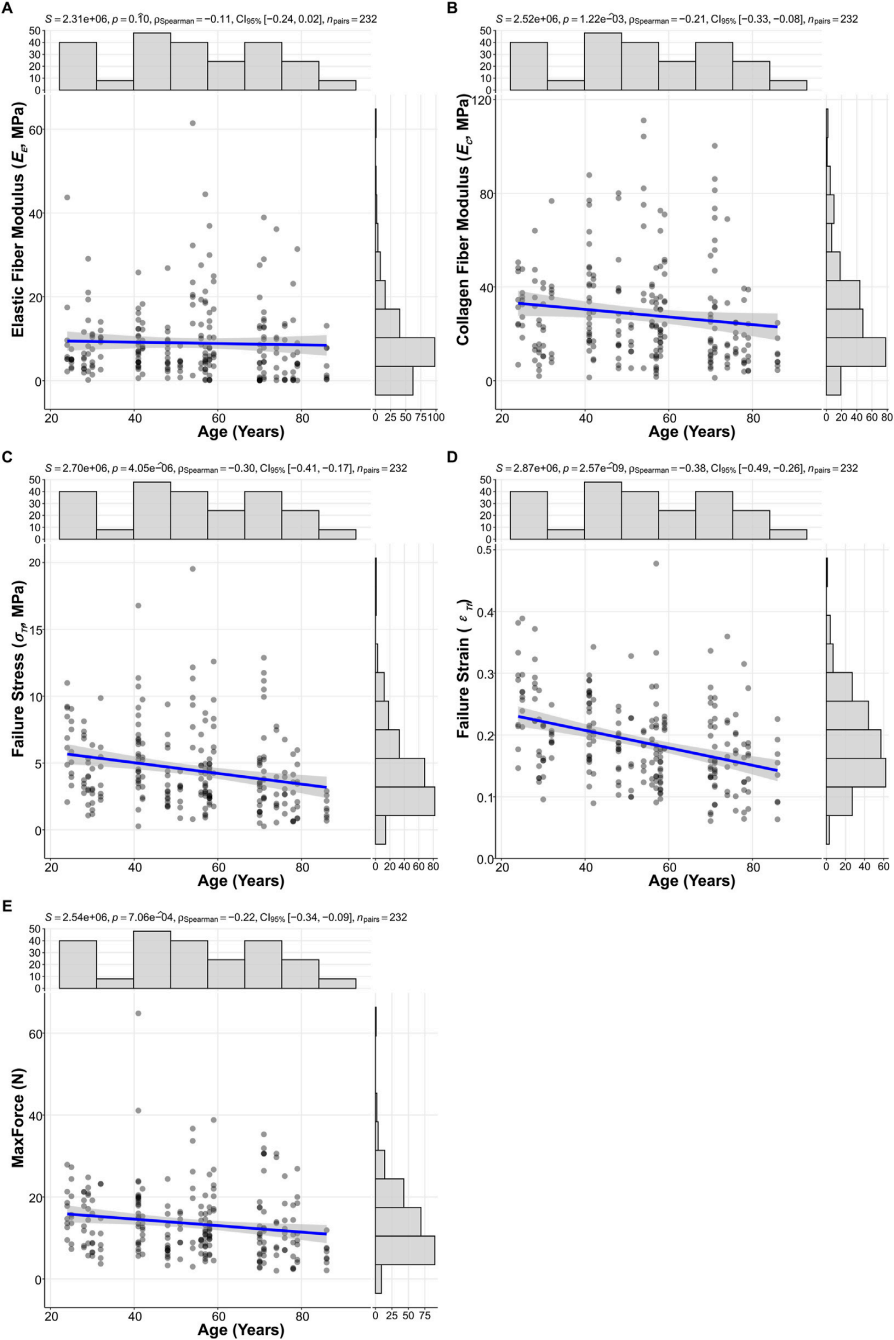
Scatterplots illustrating age-related changes in **(A)** Elastic fiber modulus (*E*_*E*_), **(B)** Collagen fiber modulus (*E*_*C*_), **(C)** Failure stress (*σ*_*Tf*_), **(D)** Failure strain (*ε*_*Tf*_), and **(E)** ultimate tensile force (MaxForce). Spearman correlation coefficients (*ρ*), p-values, and 95% confidence intervals (CI 95%) are displayed for each relationship (N = 232).

The primary fitted model parameters (K, A, B) were utilized to derive the elastic fiber modulus (*E*_*E*_) and collagen fiber modulus (*E*_*C*_) for each specimen, allowing for an assessment of age-dependent changes across the defined cohorts. Analysis of these moduli and the ultimate mechanical properties revealed distinct age-dependent patterns for some parameters, while others showed no clear overall age-related trends (Figure 4). The *E*_*E*_ did not show a statistically significant correlation with age (Figure 4A; Spearman’s correlation coefficient *ρ* = −0.11, P = 0.10). In contrast, the *E*_*C*_ exhibited a significant decrease with advancing age. For example, *E*_*C*_ declined from 28.0 (15.4, 40.8) MPa in the young adult cohort to 15.3 (9.11, 29.0) MPa in the elderly cohort (Figure 4B; *ρ* = −0.21, P = 1.22 × 10^−3^).

Ultimate tensile properties also demonstrated significant age-related declines. Notably, failure strain (*ε*_*Tf*_) showed the most pronounced degradation, significantly decreasing with age (Figure 4D; *ρ* = −0.38, P = 2.57 × 10^−9^). This was evidenced by the decline in *ε*_*Tf*_ 0.215 (0.167, 0.269) in the young adult cohort, to 0.175 (0.132, 0.211) in the middle-aged cohort, and further to 0.156 (0.118, 0.202) in the elderly cohort. Additionally, failure stress *σ*_*Tf*_ (Figure 4C; *ρ* = −0.30, P = 4.05 × 10^−6^), and MaxForce (Figure 4E; *ρ* = −0.22, P = 7.06 × 10^−4^) also showed significant decreases with advancing age.

### 3.4 Regional and directional variations in dura material properties

#### 3.4.1 Regional analysis

**FIGURE 5:**
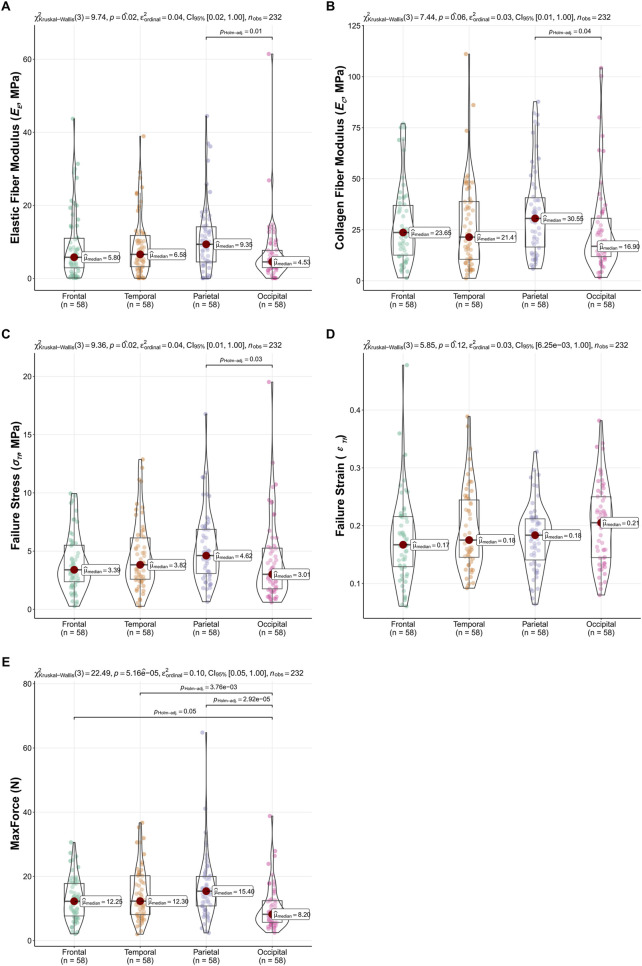
Regional variations in dura mater material properties. Box-Violin plots illustrating **(A)** Elastic Fiber Modulus (*E*_*E*_), **(B)** Collagen Fiber Modulus (*E*_*C*_), **(C)** Failure Stress (*σ*_*Tf*_), **(D)** Failure Strain (*ε*_*Tf*_), and **(E)** MaxForce across frontal, temporal, parietal, and occipital regions. p-values from overall Kruskal–Wallis tests are indicated, and significant pairwise differences (Dunn’s test, P < 0.05) are highlighted.

The influence of anatomical region (frontal, temporal, parietal, occipital) on the biomechanical properties of the dura mater was investigated using Kruskal–Wallis tests, with results summarized in Figure 5.

Significant regional differences were observed for the *E*_*E*_ (P = 0.02), *σ*_*Tf*_ (P = 0.02), and MaxForce (P < 0.001). Dunn’s multiple comparisons for *E*_*E*_ revealed significantly higher values in the parietal region compared to the occipital region (P = 0.01). For *σ*_*Tf*_, the parietal region also showed significantly higher values than the occipital region (P = 0.03). MaxForce exhibited the most widespread regional variation, with the occipital region demonstrating significantly lower values compared to the frontal (P = 0.05), temporal (P < 0.001), and parietal (P < 0.001) regions. The *E*_*C*_ showed a trend towards regional differences (P = 0.059), with pairwise comparisons indicating higher *E*_*C*_ in the parietal region compared to the occipital region (P = 0.04). No significant regional variations were found for *ε*_*Tf*_ (P = 0.12). These regional comparisons are visually detailed in Figure 5.

#### 3.4.2 Directional analysis

The effect of loading direction (coronal and sagittal) on material properties was also assessed (Table 3). Significant differences between loading directions were found for *E*_*C*_ (P = 0.003), *σ*_*Tf*_ (P = 0.020), and MaxForce (P = 0.014). For all three parameters showing significant directional dependence, testing in the sagittal direction yielded higher median values compared to the coronal direction. Specifically, median *E*_*C*_ was 27.0 (14.9, 41.4) MPa in the sagittal direction versus 18.1 (10.5, 31.6) MPa in the coronal direction. Median *σ*_*Tf*_ was 4.30 (2.66, 6.16) MPa sagittal versus 3.18 (2.29, 5.09) MPa coronal. Median MaxForce was 12.9 (8.65, 19.1) N sagittal versus 10.3 (7.20, 16.1) N coronal.

**TABLE 3:** Comparison of dura mater material properties between coronal and sagittal tensile directions.

Parameter	Direction	N	Median	IQR	25th percentile	75th percentile	Kruskal–Wallis χ^2^ (df = 1)	P value
*E*_*E*_ (MPa)	Coronal	116	5.45	6.97	3.15	10.1	2.58	0.108
	Sagittal	116	7.52	9.74	3.41	13.1		
*E*_*C*_ (MPa)	Coronal	116	18.1	21.1	10.5	31.6	8.93	0.003*
	Sagittal	116	27.0	26.5	14.9	41.4		
*σ*_*Tf*_ (MPa)	Coronal	116	3.18	2.80	2.29	5.09	5.38	0.02*
	Sagittal	116	4.30	3.50	2.66	6.16		
*ε*_*Tf*_	Coronal	116	0.185	0.0822	0.145	0.227	0.724	0.395
	Sagittal	116	0.177	0.0876	0.133	0.221		
MaxForce (N)	Coronal	116	10.3	8.88	7.20	16.1	6.03	0.014*
	Sagittal	116	12.9	10.4	8.65	19.1		

*Note: Data are presented as Median (Interquartile Range, IQR).* indicates significant p-values (P < 0.05).

No statistically significant differences between loading directions were observed for *E*_*E*_ (P = 0.108) or *ε*_*Tf*_ (P = 0.395). Detailed descriptive statistics and Kruskal–Wallis test results for directional comparisons are presented in Table 3.

### 3.5 Influence of sex on dural material properties

To investigate the influence of sex on the material properties of the dura mater, data from 144 male and 88 female specimens were analyzed across four anatomical regions (frontal, temporal, parietal, and occipital). Overall comparisons of *E*_*E*_, *E*_*C*_, *σ*_*Tf*_, and *ε*_*Tf*_ between male and female groups were conducted using Kruskal–Wallis tests. The results revealed that no statistically significant differences between sexes for the evaluated material properties: *E*_*E*_ (P = 0.266), *E*_*C*_ (P = 0.551), *σ*_*Tf*_ (P = 0.133), and *ε*_*Tf*_ (P = 0.626). Detailed descriptive statistics are presented in Table 4.

**TABLE 4:** Descriptive statistics of dura mater material properties stratified by sex and anatomical region.

Sex	Region	N	*E*_*E*_ (MPa)	*E*_*C*_ (MPa)	*σ*_*Tf*_ (MPa)	*ε*_*Tf*_
Male	Frontal	36	6.56 (2.81, 11.6)	24.1 (14.8, 40.6)	4.02 (2.66, 5.87)	0.167 (0.131, 0.217)
	Temporal	36	7.54 (3.89, 12.3)	16.5 (8.54, 40.1)	3.89 (2.37, 6.24)	0.175 (0.143, 0.230)
	Parietal	36	9.80 (4.19, 17.1)	29.8 (18.1, 47.1)	4.53 (3.23, 7.22)	0.186 (0.149, 0.211)
	Occipital	36	4.73 (3.07, 9.30)	18.4 (14.4, 31.9)	3.26 (1.94, 5.63)	0.209 (0.157, 0.236)
	Total	144	6.60 (3.19, 12.5)	23.8 (12.8, 39.7)	3.97 (2.41, 6.41)	0.183 (0.144, 0.224)
Female	Frontal	22	5.42 (2.93, 10.3)	19.9 (9.57, 33.7)	2.90 (1.93, 3.69)	0.174 (0.113, 0.214)
	Temporal	22	5.47 (3.02, 10.3)	30.4 (15.9, 38.8)	3.70 (3.04, 5.77)	0.173 (0.150, 0.270)
	Parietal	22	9.07 (5.08, 12.7)	31.3 (14.6, 38.8)	4.74 (2.86, 5.65)	0.175 (0.122, 0.212)
	Occipital	22	4.45 (1.74, 6.54)	14.0 (10.6, 26.3)	2.73 (1.25, 4.46)	0.182 (0.138, 0.255)
	Total	88	5.77 (3.10, 10.9)	22.0 (11.2, 35.7)	3.43 (2.30, 5.09)	0.173 (0.131, 0.229)

Note: Data are presented as Median (Interquartile Range, IQR).

Interestingly, while overall statistical significance was not reached, examination of the total median values (Table 4) indicated a descriptive trend where males exhibited slightly higher median values than females for all four material properties: *E*_*E*_ (Male: 6.60 MPa vs. Female: 5.77 MPa), *E*_*C*_ (Male: 23.8 MPa vs. Female: 22.0 MPa), *σ*_*Tf*_ (Male: 3.97 MPa vs. Female: 3.43 MPa), and *ε*_*Tf*_ (Male: 0.183 vs. Female: 0.173). However, these global descriptive trends did not translate into statistically significant differences, and regional variations (as seen in Table 4, e.g., *E*_*C*_ in the temporal region) were also present. These findings suggest that within the current cohort and statistical power, sex does not appear to be a primary determinant of these intrinsic material properties when assessed globally, although subtle descriptive trends might warrant further investigation in studies with larger sample sizes specifically powered for such comparisons (Walsh et al., 2018).

### 3.6 Histological characterization of age-related microstructural changes

**FIGURE 6:**
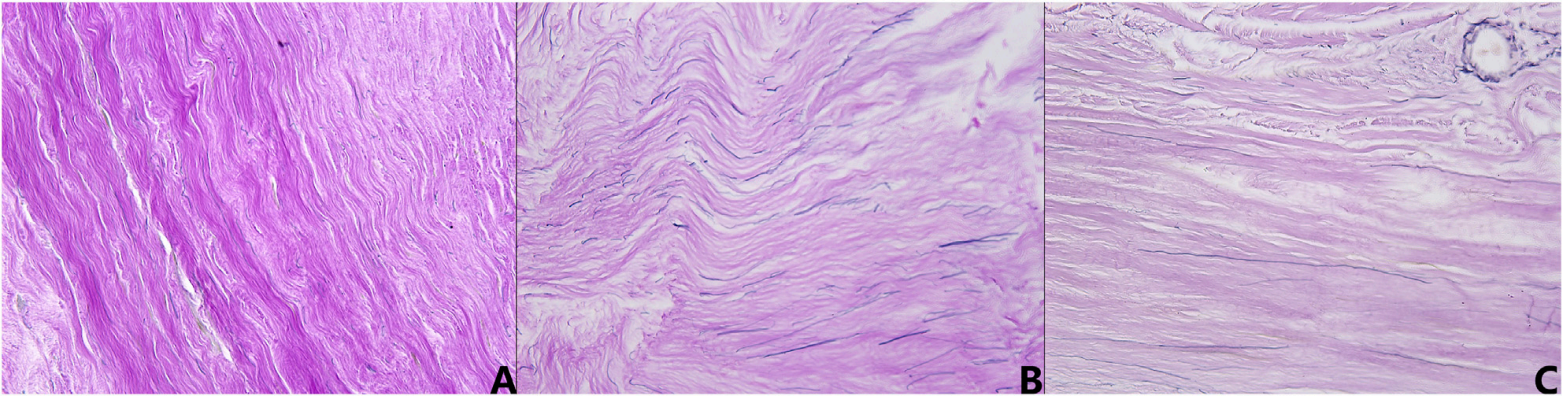
Representative micrographs of human dura mater demonstrating age-related histological changes (Verhoeff-Van Gieson stain, ×400 magnification). **(A)** Young adult specimen, showing dense, well-organized, wavy collagen bundles (red) and intact, parallel elastic fibers (blue-black). **(B)** Middle-aged specimen, exhibiting less compact collagen, with some reduction in fiber robustness and more prominent elastic fibers. **(C)** Elderly specimen, displaying a more amorphous collagen matrix, loss of distinct fiber waviness, and fragmented, reduced elastic fibers.

Representative histological sections from the young adult, middle-aged, and elderly cohorts were qualitatively assessed using Verhoeff-Van Gieson staining (collagen red, elastic fibers blue-black) to examine age-related microstructural changes (Figure 6). Young adult specimens (Figure 6A) demonstrated a dense, well-organized collagenous matrix with robust, wavy fiber bundles. Interspersed elastic fibers were clearly discernible, oriented parallel to collagen, forming an intact network. Middle-aged specimens (Figure 6B) showed initial microstructural alterations. Collagen staining appeared somewhat diminished, fibers seemed less coarse, and their arrangement was less compact. While elastic fibers remained largely parallel to collagen, their network appeared less uniform and, in some areas, relatively more prominent. Elderly specimens (Figure 6C) exhibited the most pronounced changes. The collagenous matrix often appeared paler, losing its organized fibrous character and becoming more amorphous or homogenized, with diminished fiber waviness. Elastic fibers were notably reduced, fragmented, and their organized arrangement was frequently disrupted. These observations indicate progressive, age-associated degradation and disorganization of dural fibrous components.

## 4 Discussion

### 4.1 Importance of population-specific dural characterization for biomechanical modeling

The human dura mater, a critical component of the intracranial protective system, not only provides mechanical support but also influences cerebrospinal fluid circulation and venous drainage. Its biomechanical integrity is paramount, as studies like those by Cavelier et al. (2022b) have shown that dural attachment significantly alters skull properties, such as bending strength and elastic modulus. Consequently, accurate representation of dural material properties is essential for the biofidelity of computational tools like finite element models used in traumatic brain injury research and forensic investigations (Zwirner et al., 2023). However, a significant gap exists in population-specific data, particularly for the Chinese demographic. Widely utilized FE head models (e.g., THUMS (I et al., 2002), GHBMC (Mao et al., 2013), KTH (Kleiven and Hardy, 2002)) often employ a generic elastic modulus (31.5 MPa) for the dura mater, largely neglecting inter-individual variability, including crucial age-related and regional differences (Meagher and Tiernan, 2020). This simplification can compromise the predictive accuracy of these models regarding injury thresholds and mechanisms. The present study addresses this deficit by providing comprehensive, population-specific biomechanical data for the Chinese adult dura mater, aiming to enhance the precision of FE models and ultimately contribute to more accurate forensic TBI analysis and clinical assessments.

Given the composite nature of the dura mater, which is predominantly composed of collagen fibers, with a smaller, yet functionally significant, elastin component, this study employed the microstructurally-based constitutive model proposed by Raghavan et al. (2011). This approach was chosen to distinctly characterize the contributions of collagen and elastic fibers to the tissue’s overall mechanical response, specifically their respective moduli. By isolating these components, the model aims to provide a more nuanced understanding of the tissue’s behavior compared to bulk phenomenological models and can offer more specific parameters for advanced FE simulations that account for fibrous contributions.

### 4.2 Regional heterogeneity of dural biomechanical properties

The observed regional heterogeneity in dural biomechanical properties is critical for enhancing the biofidelity of computational models used in traumatic brain injury (TBI) research and forensic analyses (Jia et al., 2020). Our study identified some variations across the frontal, temporal, parietal, and occipital regions of dura mater. Notably, the parietal region consistently demonstrated superior mechanical characteristics, exhibiting significantly higher *E*_*E*_ (P = 0.01) and *σ*_*Tf*_ (P = 0.03) compared to the occipital region, with a similar trend for *E*_*C*_ (P = 0.04). The occipital region, in contrast, generally showed the lowest mechanical resilience, particularly in MaxForce (P < 0.001 vs. other regions). However, *ε*_*Tf*_ did not differ significantly across regions (P = 0.12), and for *E*_*E*_, *E*_*C*_, and *σ*_*Tf*_, many pairwise comparisons between other regions also did not reach statistical significance. These findings present a nuanced picture. De Kegel et al. (2018) reported no significant regional differences in material properties for human cranial dura, suggesting that the dura could be modeled as regionally homogeneous for global mechanical response. Similarly, while Niestrawska et al. (2023) reported the dorsal-medial part of human dura often possesses higher failure stress compared to the lateral region (5.06 (4.49, 6.83) MPa and 2.51 (1.73, 3.15) MPa, P = 0.046), their data also show considerable overlap in property ranges between different broader regions for certain metrics. Other studies, often in animal models or focusing on specific features like the superior sagittal sinus, have reported more pronounced regional variations (Walsh et al., 2018; Walsh et al., 2021).

Considering our results in conjunction with the literature, it appears that while the human dura mater is not entirely uniform, the extent of regional variation may be parameter-dependent and potentially less pronounced for bulk tensile properties across some major cranial expanses than previously assumed. For FE modeling purposes, this could imply that a largely homogeneous material assignment for the dura might be a reasonable simplification for many applications, particularly those focused on global brain response. However, the statistically significant differences we observed, especially the distinct mechanical behavior of the parietal and occipital regions in terms of *E*_*E*_, *E*_*C*_, and *σ*_*Tf*_, should not be entirely disregarded. For high-fidelity models, or when investigating injury mechanisms specifically implicating these regions (parietal impacts or occipital contrecoup injuries), incorporating these specific regional property adjustments would be advisable. Future research could further clarify the functional implications of these specific parietal-occipital differences.

### 4.3 Anisotropic mechanical behavior of the dura mater

The dura mater is recognized as an anisotropic material, primarily due to the organized arrangement of its collagen fibers (Balandin et al., 2022; Protasoni et al., 2011; van Noort et al., 1981). Our investigation into the directional dependence of biomechanical properties in Chinese adult dura mater, comparing sagittal and coronal loading directions, supports this anisotropic nature. Statistically significant differences between the sagittal and coronal directions were found for the *E*_*C*_ (P = 0.003), *σ*_*Tf*_ (P = 0.02), and MaxForce (P = 0.014). Notably, for all three of these parameters, testing in the sagittal direction yielded significantly higher median values compared to the coronal direction. Specifically, median *E*_*C*_ was 27.0 MPa (sagittal) versus 18.1 MPa (coronal), median *σ*_*Tf*_ was 4.30 MPa (sagittal) versus 3.18 MPa (coronal), and median MaxForce was 12.9 N (sagittal) versus 10.3 N (coronal). This indicates that the dura mater exhibited greater stiffness in the collagen-dominated phase and higher tensile strength when loaded along the sagittal axis. Conversely, no statistically significant directional differences were observed for the *E*_*E*_ (P = 0.108) or the *ε*_*Tf*_ (P = 0.395), suggesting that the initial elastic response and the ultimate stretchability before rupture were comparable between these two orthogonal directions.

These findings of sagittal direction exhibiting superior strength and stiffness are consistent with observations in human spinal dura mater, where Cavelier et al. (2022a) reported significantly higher strength and stiffness in the longitudinal (analogous to cranial sagittal) direction compared to the circumferential (analogous to cranial coronal) direction. For human cranial dura, Niestrawska et al. (2023) provided detailed regional and directional mapping, clearly demonstrating anisotropy. Their results often indicate that mechanical properties are higher when aligned with dominant fiber bundles, such as along the superior sagittal sinus (predominantly sagittal orientation), compared to transverse orientations. Walsh et al. (2021) also confirmed the anisotropic nature of human cranial dura. The dominant sagittal mechanical performance found in our study likely reflects a more effective load-bearing architecture of collagen fibers along this axis within the tested samples, even within the complex multi-layered, interwoven network structure of the dura (Protasoni et al., 2011; Ünal and Sezgin, 2021). The demonstration of significant anisotropy has direct implications for the biofidelity of finite element models. While isotropic material models are simpler to implement, they may not accurately capture the stress-strain response of the dura under complex loading conditions. Our results underscore the importance of incorporating anisotropic constitutive laws for the dura mater in advanced FE simulations of TBI, particularly when the predicted injury mechanisms are sensitive to directional material behavior (Meagher and Tiernan, 2020). The lack of directional dependence for *E*_*E*_ and εTf might simplify certain aspects of modeling but the significant anisotropy in *E*_*C*_ and *σ*_*Tf*_ remains a critical factor.

### 4.4 Age-related changes: linking microstructure to mechanical integrity

Our histological assessment revealed progressive, age-related degradation of the dura mater’s microarchitecture, providing a structural basis for the observed decline in its mechanical integrity. Young adult specimens consistently showed a dense, well-organized collagenous framework with intact elastic fiber networks. Indeed, this structural deterioration progressed with age, from initial alterations in middle-aged samples to more profound disorganization in the elderly, notably featuring a more amorphous collagen matrix. These qualitative observations of microstructural decay are consistent with previous reports on age-related dural changes (Li et al., 2020; Balandin et al., 2022; Mingjie et al., 2020). These age-related decreases in dural failure stress and strain could directly influence a forensic interpretation, which predicts high risk of dural tearing in elderly versus younger individuals in forensic cases.

The observed degeneration of the collagen network—the primary load-bearing component of the dura (Protasoni et al., 2011; Fung, 1993)—directly correlates with the significant age-dependent reductions in *E*_*C*_, *σ*_*Tf*_, and MaxForce. As collagen fibers lose their organization, density, and potentially intrinsic quality, the tissue’s capacity to resist tensile loads and its overall stiffness at higher strains are compromised. Furthermore, the pronounced decrease in *ε*_*Tf*_ with age, indicating increased brittleness, likely reflects the combined effect of degraded collagen and compromised elastic fiber function, leading to a reduced ability of the tissue to stretch before rupture. Interestingly, despite the clear histological evidence of elastic fiber reduction and fragmentation in elderly specimens, the *E*_*E*_ did not show a statistically significant correlation with age in our cohort. This suggests that the macroscopic initial stiffness, as characterized by *E*_*E*_ in our model, is not solely dictated by the visible quantity or apparent integrity of elastic fibers. Several factors could contribute to this observation. The remaining elastic fibers, though fewer, might undergo compositional or structural alterations that affect their individual mechanical behavior, or the initial tensile response could be increasingly influenced by the pre-tension or earlier engagement of the less wavy, disorganized collagen network in aged tissue. It is also possible that the inherent variability in *E*_*E*_ measurements, or limitations in the model’s ability to perfectly decouple elastic and collagen contributions in highly degraded tissue, mask a subtle underlying trend.

### 4.5 Influence of sex on dural material properties

The potential influence of sex on the biomechanical properties of human dura mater has been a subject of investigation, though findings in the literature remain inconclusive. In our study, analysis across four anatomical regions revealed no statistically significant differences between male and female specimens for any of the evaluated material properties: *E*_*E*_, *Ec*, *σ*_*Tf*_, and *ε*_*Tf*_ when assessed globally (P > 0.05 for all). This lack of statistically significant sex-based differentiation in dural biomechanics aligns with findings from several previous studies. Li et al. (2020) and Mingjie et al. (2020) reported no significant sex differences for the tensile properties of Chinese frontal dura mater, and Zwirner et al. (2019) similarly found no significant influence of sex on the mechanical behavior of temporal dura mater in their cohort. While our data did indicate a slight, non-significant descriptive trend towards higher median values in males for all measured parameters (e.g., median *σ*_*Tf*_ Male: 3.97 MPa vs. Female: 3.43 MPa), these subtle variations did not translate to statistical significance within the scope and power of the current study. The absence of sex-related differences suggests that sex may not be a primary determinant of the dural material properties. For development of finite element models of the head, these results imply that sex-specific parameterization of the dura mater may not be critical for capturing its bulk mechanical response, potentially simplifying model development. However, it is acknowledged that subtle differences might exist, and studies with larger sample sizes could further clarify the potential minor sex-based distinctions that are present (Raghavan et al., 2011). For now, other factors such as age and potentially anatomical region appear to exert a more dominant influence on dural biomechanics than sex.

### 4.6 Limitations and future directions

While the present study provides a comprehensive characterization of Chinese adult dura mater, certain limitations inherent to such research should be noted, alongside avenues for future investigation. Our study utilized 232 specimens from 29 donors, a sample size that is substantial compared to many existing reports on dural biomechanics (Kizmazoglu et al., 2019; Pearcy et al., 2022; Cavelier et al., 2022a; Niestrawska et al., 2023; Zwirner et al., 2023). However, human biological tissues exhibit considerable inherent variability. Therefore, while our findings on age, region, and direction provide insights for this cohort, further expanding the donor pool, potentially through multi-center cohorts, would be invaluable for detecting more subtle influencing factors and for enhancing the generalizability of nuanced statistical trends, especially concerning the full spectrum of population-wide variability. The findings are specific to the Chinese adult population. While this provides crucial baseline data, comparative studies across different ethnic groups would be necessary to understand potential population-specific dural characteristics, though such endeavors often require extensive collaborative efforts.

Methodologically, we employed rigorous inclusion/exclusion criteria for sample selection and standardized protocols for specimen preparation, including efforts to maintain tissue hydration during testing. Nevertheless, unrecorded antemortem or peri-mortem factors (such as specific causes of death) could contribute to data scatter. Our characterization of anisotropy was based on testing along sagittal and coronal axes, and mechanical testing was conducted under quasi-static conditions. Future investigations incorporating biaxial tensile testing and dynamic, high strain-rate loading would offer a more complete biomechanical profile pertinent to complex injury scenarios. Similarly, while our qualitative histological analysis provided valuable insights into age-related microstructural changes, future quantitative stereological analyses would be beneficial to establish more precise correlations between specific microstructural parameters and observed macroscopic mechanical properties. Additionally, while the DIC system provided high-resolution displacement data, a formal quantification of the overall strain measurement error margin for this specific experimental setup was not performed, though standard calibration and operational procedures were strictly followed.

Finally, this study focused on the fundamental biomechanical characterization of the dura mater. The rich dataset generated herein provides a critical foundation for developing high-fidelity, population-specific finite element models and for refining forensic interpretations of head trauma. The detailed application of these data in advanced computational modeling and specific forensic case simulations represents an important subsequent phase of research.

## 5 Conclusion

This study comprehensively characterized Chinese adult dura mater, revealing significant age-related degradation in dural integrity, evidenced by a decline in collagen fiber modulus, failure stress, failure strain, which correlated with histological deterioration; elastic modulus remained age-independent. Clear anisotropy was demonstrated, with superior mechanical performance (collagen fiber modulus, failure stress) in the sagittal versus coronal direction. Regional variations were also evident, notably stronger parietal dura compared to occipital. In contrast, no significant sex-related differences were found. These population-specific data on age, directional, and regional dependencies provide crucial parameters to enhance the biofidelity of finite element models and refine forensic investigations. This research significantly advances the understanding of dura mater biomechanics and provides a valuable dataset for future clinical and forensic applications.

## Data Availability

The raw data supporting the conclusion of this article will be made available by the authors, without undue reservation.
