# Supplementary material for: Uniaxial tensile material properties of adult Chinese dura mater: investigating the influence of age, sex, and anatomical site
Source: Front Bioeng Biotechnol. 2025 Jul 29;13:1550228. doi: 10.3389/fbioe.2025.1550228 (PMC12339512; doi:10.3389/fbioe.2025.1550228)
Supplement: Supplementary file 1 [file Table1.docx]

Supplementary Table 1. Summary of the donors’ information and the thickness of specimens of dura mater

| Case | Sex | Age | Sagittal | | | | Coronal | | | |
| --- | --- | --- | --- | --- | --- | --- | --- | --- | --- | --- |
|  |  |  | F | T | P | O | F | T | P | O |
| C1 | M | 24 | 0.42 | 0.42 | 0.44 | 0.60 | 0.40 | 0.51 | 0.75 | 0.59 |
| C2 | F | 25 | 0.84 | 0.6 | 0.63 | 0.40 | 0.87 | 0.6 | 0.58 | 0.43 |
| C3 | F | 28 | 0.73 | 0.6 | 0.61 | 0.51 | 0.79 | 0.65 | 0.59 | 0.36 |
| C4 | M | 29 | 0.92 | 0.79 | 0.88 | 0.87 | 0.89 | 0.97 | 0.82 | 1.12 |
| C5 | F | 30 | 0.58 | 0.72 | 0.58 | 0.89 | 0.53 | 0.64 | 0.68 | 0.76 |
| C6 | F | 32 | 0.59 | 0.49 | 0.45 | 0.35 | 0.54 | 0.47 | 0.50 | 0.34 |
| C7 | M | 41 | 0.67 | 0.57 | 0.71 | 0.43 | 0.78 | 0.67 | 0.92 | 0.42 |
| C8 | M | 41 | 0.53 | 0.51 | 0.60 | 0.50 | 0.58 | 0.45 | 0.55 | 0.45 |
| C9 | M | 41 | 0.50 | 0.48 | 0.44 | 0.46 | 0.67 | 0.61 | 0.56 | 0.52 |
| C10 | M | 42 | 0.43 | 0.47 | 0.54 | 0.46 | 0.48 | 0.42 | 0.62 | 0.65 |
| C11 | M | 48 | 0.5 | 0.9 | 0.48 | 0.44 | 0.51 | 0.50 | 0.42 | 0.32 |
| C12 | M | 48 | 0.54 | 0.4 | 0.48 | 0.36 | 0.49 | 0.56 | 0.65 | 0.44 |
| C13 | M | 51 | 0.78 | 0.89 | 0.89 | 0.71 | 0.81 | 0.95 | 0.86 | 0.88 |
| C14 | M | 54 | 0.49 | 0.72 | 0.44 | 0.52 | 0.53 | 0.56 | 0.57 | 0.49 |
| C15 | M | 56 | 0.42 | 0.42 | 0.53 | 0.47 | 0.56 | 0.45 | 0.43 | 0.48 |
| C16 | M | 57 | 0.80 | 0.92 | 0.86 | 0.89 | 0.89 | 0.75 | 0.69 | 0.91 |
| C17 | F | 57 | 0.67 | 0.46 | 0.53 | 0.41 | 0.53 | 0.49 | 0.55 | 0.45 |
| C18 | M | 58 | 1.02 | 0.89 | 0.74 | 0.82 | 0.87 | 0.73 | 0.90 | 0.80 |
| C19 | F | 58 | 0.45 | 0.47 | 0.53 | 0.47 | 0.42 | 0.43 | 0.54 | 0.51 |
| C20 | M | 59 | 0.79 | 0.84 | 0.79 | 0.77 | 0.80 | 0.63 | 0.85 | 0.76 |
| C21 | F | 70 | 0.65 | 0.72 | 0.64 | 0.63 | 0.55 | 0.61 | 0.53 | 0.73 |
| C22 | M | 70 | 0.89 | 0.64 | 0.54 | 0.43 | 0.53 | 0.75 | 0.49 | 0.79 |
| C23 | F | 71 | 0.43 | 0.44 | 0.44 | 0.43 | 0.45 | 0.45 | 0.45 | 0.44 |
| C24 | M | 71 | 0.47 | 0.46 | 0.44 | 0.44 | 0.51 | 0.46 | 0.45 | 0.46 |
| C25 | M | 74 | 0.82 | 0.64 | 0.81 | 0.60 | 1.02 | 0.64 | 0.75 | 0.81 |
| C26 | M | 76 | 0.90 | 0.7 | 0.93 | 0.64 | 1.03 | 0.57 | 0.70 | 0.57 |
| C27 | F | 78 | 0.71 | 0.72 | 0.54 | 0.77 | 0.63 | 0.56 | 0.64 | 0.67 |
| C28 | F | 79 | 0.86 | 0.71 | 0.8 | 0.86 | 0.95 | 0.88 | 0.80 | 0.82 |
| C29 | F | 86 | 0.57 | 0.59 | 0.68 | 0.80 | 0.60 | 0.72 | 0.81 | 0.82 |

Note: Sex (M = male, F = female) and regions ( F=Frontal, T=Temporal, P=Parietal, O=Occipital).
